# Sleep Phenotyping in a Mouse Model of Extreme Trait Anxiety

**DOI:** 10.1371/journal.pone.0040625

**Published:** 2012-07-11

**Authors:** Vladimira Jakubcakova, Cornelia Flachskamm, Rainer Landgraf, Mayumi Kimura

**Affiliations:** Max Planck Institute of Psychiatry, Munich, Germany; University of Regensburg, Germany

## Abstract

**Background:**

There is accumulating evidence that anxiety impairs sleep. However, due to high sleep variability in anxiety disorders, it has been difficult to state particular changes in sleep parameters caused by anxiety. Sleep profiling in an animal model with extremely high vs. low levels of trait anxiety might serve to further define sleep patterns associated with this psychopathology.

**Methodology/Principal Findings:**

Sleep-wake behavior in mouse lines with high (HAB), low (LAB) and normal (NAB) anxiety-related behaviors was monitored for 24 h during baseline and recovery after 6 h sleep deprivation (SD). The amounts of each vigilance state, sleep architecture, and EEG spectral variations were compared between the mouse lines. In comparison to NAB mice, HAB mice slept more and exhibited consistently increased delta power during non-rapid eye movement (NREM) sleep. Their sleep patterns were characterized by heavy fragmentation, reduced maintenance of wakefulness, and frequent intrusions of rapid eye movement (REM) sleep. In contrast, LAB mice showed a robust sleep-wake rhythm with remarkably prolonged sleep latency and a long, persistent period of wakefulness. In addition, the accumulation of delta power after SD was impaired in the LAB line, as compared to HAB mice.

**Conclusions/Significance:**

Sleep-wake patterns were significantly different between HAB and LAB mice, indicating that the genetic predisposition to extremes in trait anxiety leaves a biological scar on sleep quality. The enhanced sleep demand observed in HAB mice, with a strong drive toward REM sleep, may resemble a unique phenotype reflecting not only elevated anxiety but also a depression-like attribute.

## Introduction

The prevalence of affective disorders increases in a global scale and has negatively contributed to serious health and social problems in recent years [1]. The psychopathology of these stress-related mental disorders includes symptoms that reflect disturbances in sleep. Long sleep latency, early awakening, and sleep fragmentation are examples of sleep characteristics that are strongly associated with depression [2]–[5]. However, in many patients with anxiety, a high degree of comorbidity with depression is found [6], [7], and the sleep problems associated with anxiety have never been clearly elucidated.

Given the considerable overlap of symptoms and causes between anxiety and depression, there is growing interest in finding reliable diagnostic markers in patients. In this respect, the potential of experimental non-human models could be of considerable help to recognize and evaluate the discrete clinical features of various psychopathologies. In fact, the exploration of sleep profiles in animal models that share relevant endophenotypes of affective disorders was shown to be useful for studying a variety of symptoms of human pathophysiologies. Altered sleep-wake patterns resembling those observed in depressed patients have been demonstrated in several rodent models of depression [8]–[15], although most studies of animal models of anxiety failed to reveal specific sleep disturbances [16], [17].

In this study, we used CD1 mice to generate an animal model with bidirectional extremes in trait anxiety. After >30 generations of selective inbreeding, mice were categorized as those showing high (HAB) or low (LAB) anxiety-related behavior on the elevated-plus maze (EPM) [18]. With selection pressure exerted on extreme anxiety phenotypes, the inbreeding protocol produced relatively homogeneous populations with enriched genetic loci relevant for the corresponding endophenotype [19]. Although bred for innate hyper-anxiety, HAB mice are also characterized by enhanced fear expression [18], [20]. Interestingly, several neuroendocrine, neuronal, and genetic impairments [21]–[26] found in rats and mice exhibiting high anxiety resemble those seen in psychiatric patients with a high degree of both anxiety and depression. In contrast, selective inbreeding for low levels of anxiety (LAB) resulted in a behavioral phenotype that is associated with reduced risk assessment and active coping style [18], [27].

The HAB/LAB mouse model, showing opposite anxiety-related and depression-like phenotypes, thus provides an opportunity for identifying sleep traits for the purposes of further refining the diagnosis of these disorders. These mice were subjected to a full array of analyses of their electroencephalographic (EEG) traces relative to findings in inbred controls showing normal anxiety-related behavior (NAB). Here, we report that EEG activities differ significantly between the different lines, with distinct sleep phenotypes being found between HAB and LAB mice that have previously not been reported for mouse models of depression and/or anxiety.

## Methods

### Experimental animals

Adult male CD1 mice weighing 25–30 g selectively inbred for high (HAB), normal (NAB) and low (LAB) anxiety-related behavior were used in our study. The mouse lines derived from the CD1 strain were inbred for extremes in trait anxiety according to their anxiety-related behavior on the elevated plus maze (EPM), with HAB mice spending <12%, NAB mice spending 30–40%, and LAB mice spending >60% on the open arms of the EPM. Passive stress coping in the forced swim and tail suspension tests, indicative of depression-like behavior, was found similarly in HAB and NAB lines and significantly lower in LABs [18]. Initially, all animals were group-housed in the breeding facility of the Max Planck Institute of Psychiatry, Munich, Germany. Later, prior to surgery, the animals were moved to a sound-attenuated recording chamber and housed individually in Plexiglas cages (25 cm×25 cm×35 cm). During recovery from surgery and throughout the experiments, the housing environment was kept at a constant temperature of 24±1°C on a 12 h light-dark cycle (lights on at 7:00 a.m.). The animals had *ad libitum* access to water and food. All animal experiments performed in the present study were conducted according to the guidance of the European Community Council Directive, and the protocol was approved by the local commission for the Care and Use of Laboratory Animals of the Government of Upper Bavaria, Germany (Az. 55.2-1-54-2531-47-06).

### Surgery

Under inhalation narcosis with an isoflurane/oxygen mixture (Isofluran, DeltaSelect, Dreieich, Germany), the mice were fixed with a stereotaxic apparatus and chronically implanted with electroencephalographic (EEG) and electromyographic (EMG) electrodes for polysomnographic recordings. The implant consisted of four gold wires (0.25–0.30 mm diameter) for EEG recordings that were placed through the skull epidurally over the frontal and parietal cortices (coordinates, A 1.5 mm and 3 mm, L ±1.7 mm each), and two additional gold wires were inserted into the cervical portion of the trapezoid muscles for EMG recordings. All electrodes were soldered to a micro-socket affixed to the skull with dental acrylic resin. Before and after the surgical operation, the animals received atropine sulphate (0.05 mg/kg, Atropine, Braun Melsungen, Melsungen, Germany) subcutaneously to stabilize circulation and meloxicam (0.5 mg/kg, Metacam, Braun Melsungen, Melsungen, Germany) as a post-operative analgesic. After surgery, the mice spent ca. two weeks for recovery in the environment mentioned above.

### Recording schedule under baseline conditions and after sleep deprivation

To monitor 24 h spontaneous sleep-wake patterns, polygraphic recordings were initiated nearly 2 weeks after the animals had received the surgical operation. The EEG recordings were performed for 24 h, beginning at 7:00 a.m., i.e., at the onset of the light period. The first day served as a baseline. On the following day, all mice were sleep-deprived by gentle handling for six consecutive hours starting at light onset (from 7:00 a.m. to 01:00 p.m.). The gentle handling procedure included the introduction of new objects into the cages to keep the animals awake [28], [29]. Any direct contact with individual animals was avoided. At the end of the deprivation period, the animals were left to sleep freely, and recording continued for the next 18 h.

### Sleep recording setups and data analysis

The lead wires of the EEG and EMG electrodes were connected to an electric swivel through a flexible tether. The weights of the swivel and the tether were counterbalanced *via* a mechanical device to near zero; thus, the mice could move almost without restriction and were easily acclimated prior to the initiation of recording. EEG and EMG signals were amplified (10,000 x), and both filtered EEGs (0.5–29 Hz, 48 dB/octave) and non-filtered EMGs that had undergone root mean square rectification were digitized by a high-speed analog-to-digital converter at a sampling rate of 64 Hz. The signals were then processed by a PC equipped with a LabVIEW program (National Instruments, Austin, TX) especially designed for sleep EEG/EMG analysis (SEA, Cologne, Germany). Polygraphic data were stored on a computer and later processed offline with the LabVIEW-based acquisition program, in which a Fast Fourier Transform (FFT) algorithm served for power spectrum analysis of particular EEG frequency contents, i.e., EEG power density within the delta (0.5–5 Hz), theta (6–9 Hz), sigma (10–15 Hz), and beta (16–23 Hz) bands. Epochs containing artifacts were eliminated from power spectral analysis. With the aid of the adapted FFT algorithm [30], spectral analysis was performed to provide semi-automatic classification of the particular vigilance states as wakefulness, rapid eye movement (REM) and non-REM (NREM) sleep that were defined in 4-s epochs. The defined vigilance states were further confirmed visually and corrected, if necessary. The EEG power density of NREM sleep specific spectra (0.5–5 Hz) was normalized by the total power averaged from all epochs that were scored as NREM sleep throughout the 24 h period across all frequency bins from 0.5 to 29 Hz, as described elsewhere [31], [32]. Sleep architecture was also analyzed with the aid of a LabVIEW-based classification program to determine whether line differences would preferentially affect either the entry of sleep episodes or their durations when calculated separately for 12 h intervals during the light and dark period, as described in our recent report [33]. Sleep fragmentation was determined by the number of transitions from one vigilance state to another in the corresponding 12 h light and dark period. Furthermore, the average latency to NREM or REM sleep was defined as the time from either the onset of the light period or the beginning of the recovery period following sleep deprivation to the appearance of the first solid NREM or REM sleep episode lasting at least 15 epochs with an allowance of 6 interrupted 4-s epochs.

### Statistics

All statistical calculations and analyses were performed using GraphPad Prism (Version 5.04, GraphPad, San Diego, CA). All results are expressed as the means ± SEM. Two-hour averaged time-course changes in the amount of each vigilance state and in delta power between lines were analyzed by two-way ANOVA with factors ‘line’ and ‘interval’. To compare ‘line’ effects on 12 h averaged amounts of vigilance states, delta power during NREM sleep, numbers and durations of sleep/wake bouts, latencies to NREM and REM sleep, numbers of transitions, and EEG power of individual frequency bands in the corresponding light or dark periods, a one-way ANOVA with a factor ‘line’ was used. The light-dark (LD) differences in sleep and wake amounts were analyzed by two-way ANOVA with factors ‘line’ and ‘LD interval’ for each vigilance state. The direct comparison between the lines of 12 h light *versus* dark dynamics in circadian amplitudes was performed by one-way ANOVA with the factor ‘line’ followed by Tukey's test. To compare effects of sleep deprivation between lines, changes in the amount of vigilance states and EEG delta power after sleep deprivation were subtracted from those during baseline recordings in 6 h intervals and evaluated by two-way ANOVA with factors ‘line’ and ‘interval’. A two-tailed *t*-test was used to determine the differences in 24 h sleep-wake patterns and delta power during baseline and recovery after sleep deprivation for each line. If the *F* values reached statistical significance, Bonferroni's adjustment was further applied for *post-hoc* analysis. *P* values <0.05 were considered statistically significant.

## Results

### Distribution of sleep and wakefulness is modified by anxiety levels

**Figure 1:**
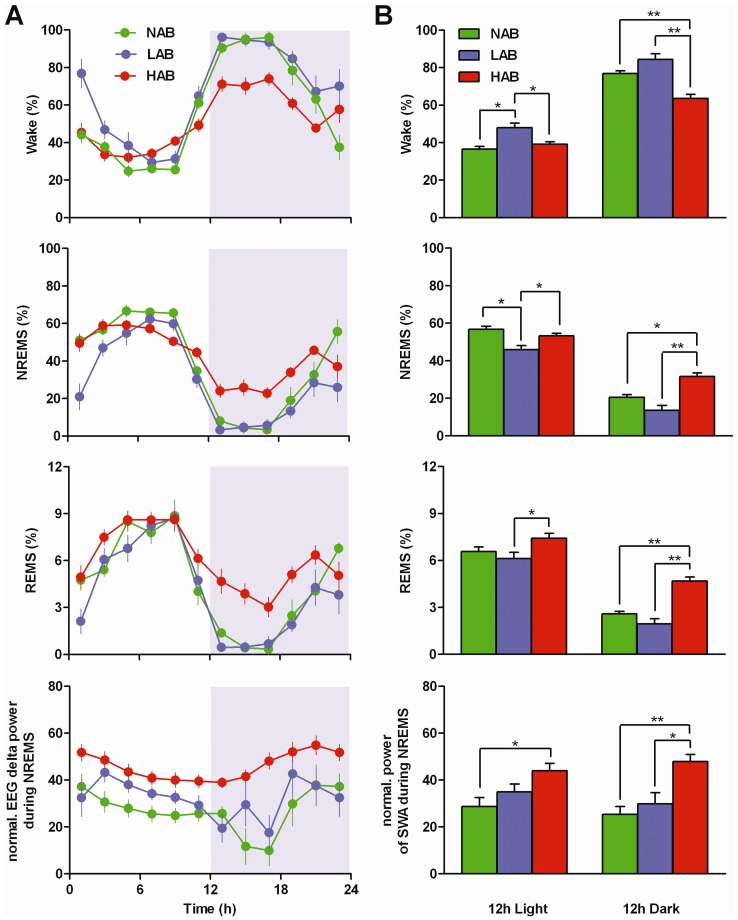
Sleep-wake distribution in the mouse model of trait anxiety. (A) Time-course changes in sleep-wake patterns and EEG delta power during NREM sleep of NAB (n = 7), LAB (n = 10) and HAB (n = 10) mice under baseline conditions. Data points represent 2 h means ± SEM of time spent in wake, NREM sleep (NREMS) and REM sleep (REMS). The delta power during NREM sleep is represented as the mean value ± SEM of normalized EEG power densities in the frequency range of 0.5–4.0 Hz restricted to NREM sleep. Two-way analysis of variance (ANOVA) revealed significant effects of ‘line’ and ‘time’ for all vigilance states and delta power (P<0.001) and their interaction for all vigilance states across 24 h (wake, P<0.001; NREMS, *P*<0.001; REMS, *P*<0.05). (B) Percentage of time spent in wake, NREM and REM sleep and the normalized EEG power of delta band during the 12 h light and dark period. Values are the 12 h means ± SEM. *P<0.05, **P<0.001, assessed by one-way ANOVA with the factor ‘line’ followed by Bonferroni's test. The light-dark (LD) differences were analyzed as well. Two-way ANOVA revealed significant effects of ‘line’ and ‘LD interval’ for all vigilance states (*P*<0.001) and their interaction for all vigilance states (*P*<0.05). The direct comparisons between lines of the 12 h LD dynamics in circadian amplitudes were performed by one-way ANOVA with the factor ‘line’ followed by Tukey's test (wake, NREMS, *P*<0.001; REMS, *P*<0.05).

All three mouse lines exhibited circadian changes in sleep-wake activity (*P*<0.001, light *versus* dark; Figure 1A). Although each line confined the majority of sleep and wakefulness to the light and dark periods, respectively, the amplitude of the light *versus* dark difference in the amount of each vigilance state was attenuated in HAB relative to NAB and LAB mice (*P*<0.05, see legend to Figure 1).

NAB mice exhibited a clear diurnal rhythm with 76.8±1.53% of the time spent awake during the dark (active) period (Figure 1B). Similarly to NAB mice, LAB mice showed a nocturnal preference for wakefulness during the dark period (Figure 1A). During the first hours of the light period, however, LAB mice exhibited increased waking activity (32.7% more than NAB, 31.5% more than HAB; *P*<0.05 each; Figure 1A). The prolonged awakenings at the start of the light period found in LAB mice contributed to the significant increase in the amount of wakefulness during the 12 h light period (11.4% more than NAB, 8.7% more than HAB; *P*<0.05 each; Figure 1B).

The amount of wakefulness in HAB mice was similar to that of NAB mice during the light period but was decreased during the dark period when compared with the two other lines (13.5% less than NAB, 20.8% less than LAB; *P*<0.001 each; Figure 1B).

NAB mice spent 56.8±1.5% of the light period in NREM sleep, and a considerable amount of NREM sleep also occurred during the dark period (20.6±1.4%, Figure 1B). LAB mice exhibited significantly reduced NREM sleep in the light period in contrast to NAB and HAB mice (10.9% less than NAB, 7.4% less than HAB; *P*<0.05 each; Figure 1B). During the light period, HAB mice spent time in NREM sleep similar to that observed in NAB mice. Throughout the dark period, however, a significant increase in NREM sleep was apparent in HAB mice (11.1% more than NAB, *P*<0.05; 18.0% more than LAB, *P*<0.001; Figure 1B).

The distribution of REM sleep in both NAB and LAB mice similarly varied across the 24 h day with less time spent in REM sleep during the dark period (Figure 1A and B). However, in HAB mice, the diurnal rhythm of REM sleep was less prominent (Figure 1A). During the light period, HAB mice showed a significantly increased level of REM sleep when compared to that of LAB mice (1.3% more, *P*<0.05; Figure 1B). Significant increases in REM sleep in HAB mice remained even through the dark period (2.1% more than NAB, 2.8% more than LAB, *P*<0.001 each; Figure 1B).

In all lines, the EEG delta power during NREM sleep progressively decreased throughout the course of the 12 h light period and increased toward the end of the dark period (Figure 1A). However, the decline in delta power in LAB mice was delayed in the light period (Figure 1A). Twelve hour means of delta power calculated in LAB mice during the light and dark period did not significantly differ from those of NAB mice (Figure 1B). Compared to those in NAB and LAB mice, constantly elevated levels of EEG delta power were detected in HAB mice throughout the 12 h light and dark periods (Figure 1A), and the averaged 12 h values in HAB mice were significantly different from those in NAB mice during the light period (15.4% more than NAB, *P*<0.05; Figure 1B) and from those in NAB and LAB mice during the dark period (22.5% more than NAB, *P*<0.001; 18.0% more than LAB, *P*<0.05; Figure 1B).

### Sleep architecture is significantly affected by different traits of anxiety

**Figure 2:**
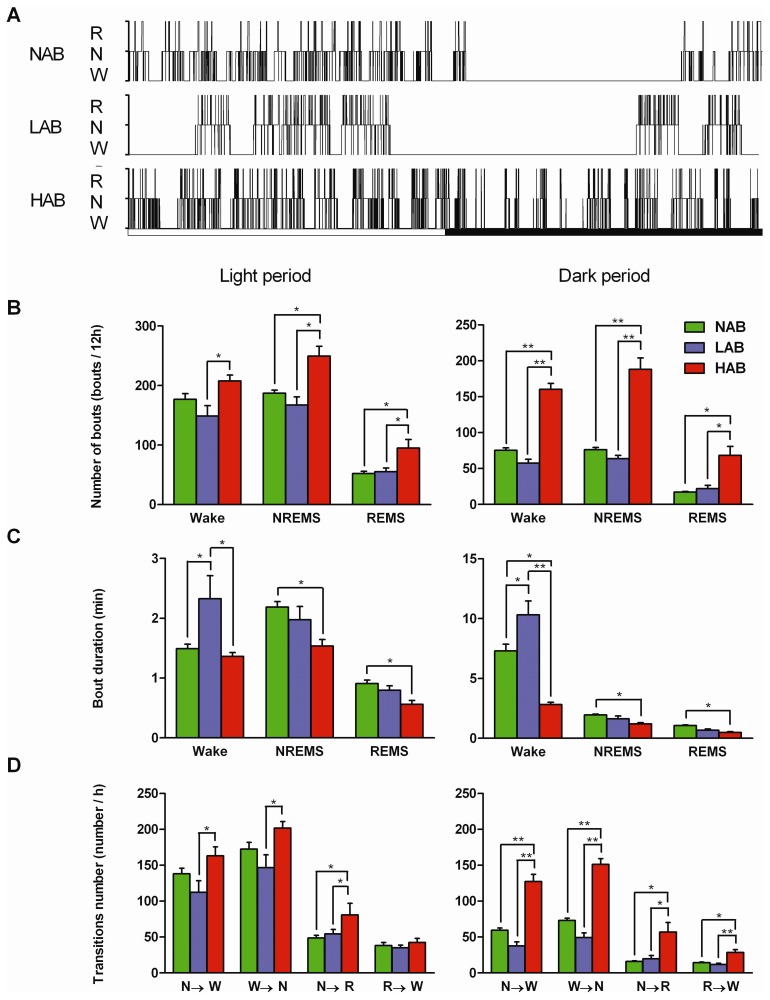
Sleep architecture of the mouse model of trait anxiety. (A) Representative 24 h hypnograms under baseline conditions of a NAB, LAB, or HAB mouse. The white-black bars below the hypnograms indicate the 12 h light-dark cycle. (W: wakefulness, N: NREM sleep, R: REM sleep) (B – D) Average numbers (B) and durations (C) of wakefulness, NREM sleep (NREMS) and REM sleep (REMS) bouts and state transitions (D) during the 12 h light and dark period. Values are the means ± SEM. NAB (n = 7), LAB (n = 10), and HAB (n = 10). Left and right panels indicate values for the light and dark periods, respectively. *P<0.05, **P<0.001, assessed by one-way ANOVA with the factor ‘line’ followed by Bonferroni's test.

As shown in the representative hypnograms (Figure 2A), NAB mice demonstrated a diurnal rhythmicity consisting of consolidated periods of sleep and wakefulness and a robust entrainment to the light-dark cycle. In contrast, marked differences in the structure, amount, and distribution of sleep and wakefulness were observed in LAB and HAB mice (Figure 2B and C).

At the beginning of the light period, LAB mice remained nearly continuously awake for the first 1 to 2 hours, thereby delaying the occurrence of the typical sleeping phase (Figure 2A). In fact, over the 12 h light period, LAB mice had longer bouts of wakefulness compared with the other two mouse lines (0.83 min/bout longer than NAB, 0.96 min/bout more than HAB; *P*<0.05 each; Figure 2C, left). Extremely long bouts of wakefulness were also evident in LAB mice during the dark period (3.0 min/bout longer than NAB, *P*<0.05; 7.5 min/bout longer than HAB, *P*<0.001; Figure 2C, right). The number of awakenings and the number or duration of NREM and REM sleep bouts did not differ across the 24 h day between LAB and NAB mice (Figure 2B and C).

Across a 24 h day, HAB mice exhibited more frequent transitions between the states of sleep and arousal than the other mouse lines (Figure 2A). During the light period, HAB mice exhibited numerous but significantly shorter NREM and REM sleep bouts and an unchanged number and length of waking bouts when compared with NAB mice (number of bouts: NREM sleep: 62.1 bouts/12 h more, REM sleep: 43.2 bouts/12 h more; duration of bouts: NREM sleep: 0.65 min/bout less, REM sleep: 0.35 min/bout less; *P*<0.05 each; Figure 2B and C, left). Further, significantly more bouts were counted for each vigilance state in HAB than in LAB mice (wake: 59.0 bouts/12 h more, NREM sleep: 82.6 bouts/12 h more, REM sleep: 39.9 bouts/12 h more, *P*<0.05 each; Figure 2B, left). The transitions from NREM sleep to wake and from wake to NREM sleep during the light period were significantly more frequent in HAB than in LAB mice (*P*<0.05, Figure 2D, left). During the dark period, HAB mice did not show a clear nocturnal preference for wakefulness when compared with the more consolidated periods of activity observed in NAB and LAB mice (Figure 2A). In contrast to the other lines, HAB mice exhibited numerous bouts of wakefulness with extremely frequent entries to NREM and REM sleep episodes (*P*<0.05 or *P*<0.001, Figure 2B and D, right). A significant reduction in the length of waking bouts was observed in HAB mice (85.0 bouts/12 h less than NAB, *P*<0.05; 102.7 bouts/12 h less than LAB, *P*<0.001; Figure 2C, right). Compared with NAB mice, episodes of NREM and REM sleep were significantly shorter in HAB mice during the dark period (NREM sleep: 0.77 min/bout shorter, REM sleep: 0.59 min/bout shorter; *P*<0.05 each; Figure 2C, right). Moreover, these alterations detected in HAB mice during the dark period were accompanied by significant increases in all state transitions when compared with the other mouse lines (*P*<0.05 or *P*<0.001; Figure 2D, right). Especially, intrusions of short REM sleep episodes were found frequently within NREM sleep across 24 h (*P*<0.05, Figure 2D). Therefore, HAB mice showed very instable state changes that were characterized as sleep fragmentation.

### Trait anxiety alters the dynamics of EEG activity during sleep

**Figure 3:**
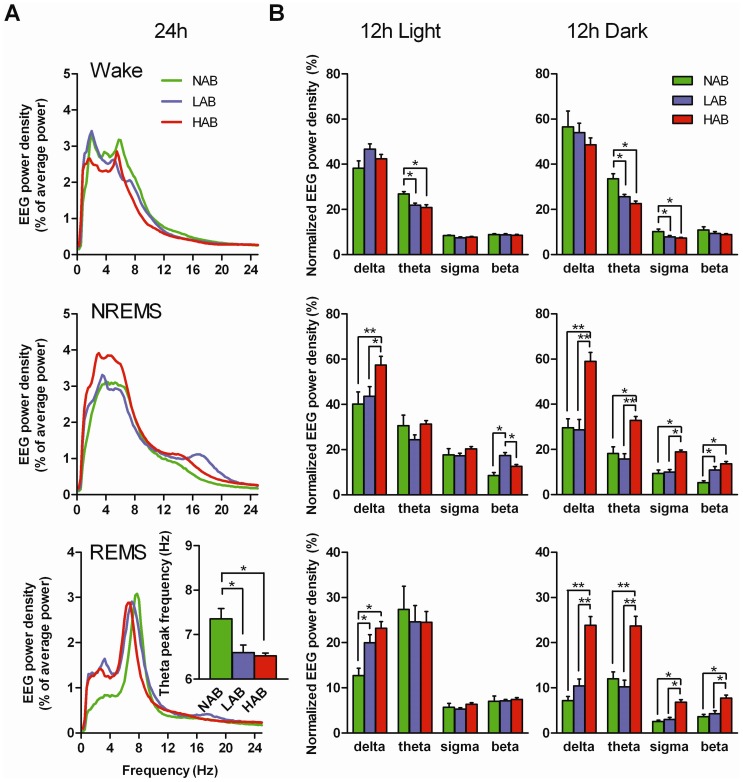
EEG spectra during wakefulness, NREM and REM sleep in the mouse model of trait anxiety. NAB (n = 7), LAB (n = 10), HAB (n = 10). (A) Spectral distribution of EEG power densities averaged from all 4-s epochs scored as wake, NREM sleep (NREMS) and REM sleep (REMS) over 24 h of recording under baseline conditions. The power of every 0.25 Hz bin was first averaged across the individual vigilance state and then normalized as a group by calculating the percentage of each bin from the total power across 24 h (0.25–32 Hz). All figures show spectral distributions of the EEG power density in the frequency range of 0.25 to 25 Hz. Two-way ANOVA with the factors ‘frequency’ and ‘line’ followed by Bonferroni's test revealed significant effects of ‘frequency’ and an interaction between ‘frequency’ and ‘line’ for each vigilance state (*P*<0.0001), whereas the ‘line’ effects were not significant. For REM sleep, the EEG theta peak frequency, between 6 and 9 Hz, is shown in the insertion. *P<0.05, assessed by one-way ANOVA with the factor ‘line’ followed by Bonferroni's test. (B) Comparisons of EEG power density within the delta (0.5–5 Hz), theta (6 – 9 Hz), sigma (10 – 15 Hz), and beta (16 – 23 Hz) bands in the 12 h light and dark period for each vigilance state. Data are presented as the 12 h means ± SEM. The EEG power of each frequency band was normalized by total EEG power during 24 h. *P<0.05, **P<0.001, assessed by one-way ANOVA with the factor ‘line’ followed by Bonferroni's test.

During wakefulness, NAB and LAB mice exhibited a higher EEG power in the broad frequency band than HAB mice (Figure 3A, top). These differences were significant either in a lower frequency range (compared to NAB: 0.75–2.25 Hz, to LAB: 1.75–2.5 Hz; *P*<0.05 each; Figure 3A) or in a range of higher frequencies (compared to NAB: 6– 8.75 Hz, to LAB: 5.75–6.5 Hz; *P*<0.05 each; Figure 3A).

In general, a spectral distribution of EEG power during NREM sleep is characterized by high values, particularly in the delta frequency band (0.5–5 Hz, Figure 3A, middle). Such an increase in power around the delta frequency during NREM sleep was more apparent in HAB mice, which reached significance in the range of 0.75–3.25 Hz when compared with NAB mice and 2.5–5.5 Hz when compared with LAB mice (*P*<0.05, Figure 3A). In LAB mice, an increase of EEG power during NREM sleep was found in the range of higher frequencies (16–17.5 Hz, *P*<0.05; Figure 3A).

During REM sleep, power densities in the theta activity (6–9 Hz) were characteristically increased in all three mouse lines. When compared with NAB mice, significant increases in REM sleep-specific power densities also occurred in LAB mice in the frequency ranges of 1–3.75 Hz and 5.5–6.75 Hz and in HAB mice in the two separate frequency ranges of 1–2.75 Hz and 5.25–8.5 Hz (*P*<0.05, Figure 3A). Moreover, the peak density within the theta range was significantly shifted toward slower frequencies in both LAB and HAB mice (Figure 3A, bottom insertion). Compared with NAB mice, the oscillations within the theta-peak frequency during REM sleep were 0.8 Hz and 0.9 Hz slower in LAB and HAB mice, respectively (*P*<0.05, Figure 3A).

Significant differences in the EEG power spectra were also evident between NAB, LAB and HAB mice across the particular frequency bands (Figure 3B).

During the light period, a significant decrease in the power of theta activity was observed in LAB and HAB mice during wakefulness (*P*<0.05, Figure 3B, top left). The power of a lower frequency range, e.g., the delta band, in HAB mice and a higher frequency range, e.g., the beta band, in LAB mice were significantly increased in NREM sleep during the light period (*P*<0.001 and *P*<0.05, respectively; Figure 3B, middle left). Compared with NAB mice, a remarkable enhancement of the power in the delta band was observed in LAB and HAB mice during REM sleep (*P*<0.05, Figure 3B, bottom left).

During the dark period, a reduction of EEG power in the theta and sigma bands during wakefulness was characteristically seen for LAB and HAB mice compared with NAB mice (*P*<0.05, Figure 3B, right). In HAB mice, the power across all four frequency bands was significantly elevated during NREM and REM sleep (*P*<0.05 or *P*<0.001, Figure 3B, right).

### Homeostatic responses to sleep deprivation (SD) are diverse in different traits of anxiety

**Figure 4:**
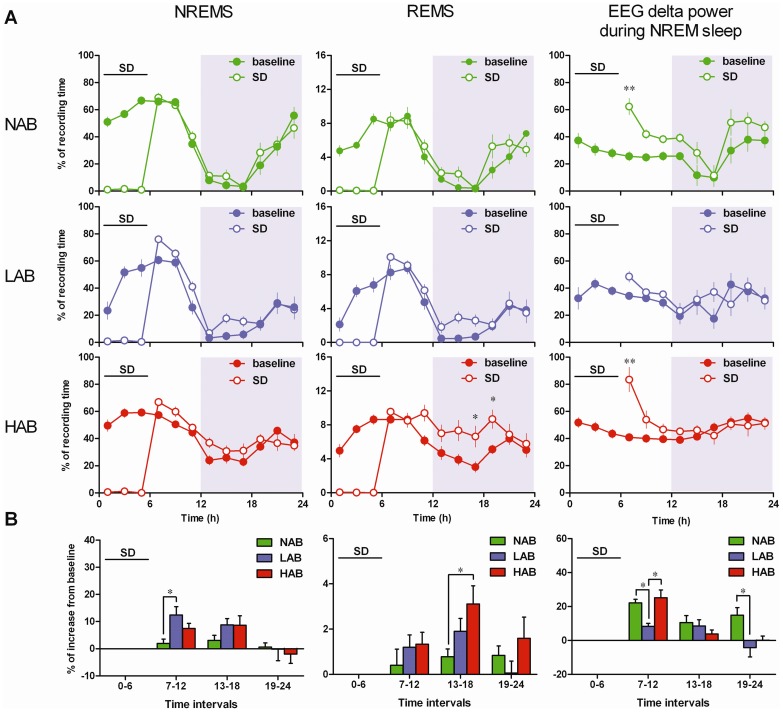
Effects of sleep deprivation on sleep recovery and EEG delta power changes during NREM sleep. (A) Time-course changes in NREM sleep (NREMS) and REM sleep (REMS) and the delta power of slow-wave activity (0.5–5 Hz) during NREM sleep are expressed as the 2 h means ± SEM during baseline recordings (closed circles) and sleep deprivation (SD, open circles) in NAB (n = 7), LAB (n = 10), HAB (n = 10) mice. SD began at the onset of the light period and lasted for 6 h. Two-way ANOVA among the three mouse lines revealed a significant effect of ‘line’ on EEG delta power in NREM sleep during the post-SD 6–12 h during the light period (*P*<0.001) and on NREM and REM sleep and EEG delta power during the post-SD 13–18 h during the dark period (*P*<0.05, *P*<0.05, *P*<0.001). * *P*<0.05, ** *P*<0.001, assessed by two-way ANOVA with the factors ‘line’ and ‘interval’ followed by Bonferroni's test.

To uncover how sleep homeostasis is controlled in the model of anxiety, mice were sleep-deprived for 6 h, and the rebound in sleep behavior was examined. Following SD, the increase of NREM sleep in the first 6 h interval (ZT 7–12) during the recovery period was significantly greater in LAB mice than in NAB mice (*P*<0.05, Figure 4B, left).

As illustrated in Figure 4A, there was an apparent enhancement of REM sleep above baseline values in HAB mice following SD, but significant increases in REM sleep were detected only between ZT 17–19 (*P*<0.05). When the differences were calculated for the second 6 h interval during the recovery period following SD, REM sleep enhancement in HAB mice was larger when compared with NAB mice (*P*<0.05, Figure 4B).

In NAB and HAB mice, there was a significant increase in EEG delta power during NREM sleep during the first 2 h of the recovery period following 6 h of forced wakefulness (*P*<0.05, Figure 4A). In contrast, the increase in EEG power density in the delta frequency range was very modest in LAB mice and did not reach significance compared with the corresponding baseline. The magnitude of increased delta power during the first 6 h interval was significantly higher in NAB and HAB than in LAB mice (*P*<0.05, Figure 4B, right). Following SD, the EEG power of the delta band decreased progressively in all lines (Figure 4B). The reduction of the power in the last 6 h of the recovery period was extreme in both LAB and HAB mice when compared with NAB mice, but the comparison was significant only between LAB and NAB mice (*P*<0.05, Figure 4B).

### Latency from wake to sleep is greatly affected by different traits of anxiety

The latencies to NREM and REM sleep from the start of the light period varied between the mouse lines (Table 1). Inter-line comparisons showed that LAB mice had an extremely prolonged latency to NREM sleep when compared with HAB mice. During baseline conditions, latency to REM sleep was significantly longer in LAB when compared with NAB and HAB mice.

**Table 1:** Latency to NREM and REM sleep during baseline conditions and after sleep deprivation in mouse models of anxiety.

		During baseline		After SD	
Line	*N*	NREMS	REMS	NREMS	REMS
NAB	7	25.6±10.0	46.7±10.4^b^	9.0±3.1	17.5±4.3^*^
LAB	10	80.2±20.9^c^	108.6±19.1^a, c^	3.3±0.5^*^	9.3±1.4^**, c^
HAB	10	12.3±7.7^b^	17.9±6.4^b^	10.5±2.5	22.0±3.1^ b^
One-way ANOVA					
*P*-value		0.0067	0.0002	0.0498	0.0124

Values are the means in min ± SEM. The letters indicate a significant between-line difference (*P*<0.05) within the individual parameter (a: from NAB mice, b: from LAB mice, c: from HAB mice), assessed by one-way ANOVA with the factor ‘line’ followed by Bonferroni's test. Statistical differences in values after sleep deprivation relative to the corresponding baseline values within each line, analyzed by *t*-test (* *P*<0.05, ** *P*<0.001). NREMS, non-REM sleep; REMS, REM sleep; SD, sleep deprivation.

Compared with baseline, 6 h of SD dramatically shortened the latencies to NREM and REM sleep in NAB and LAB mice. When calculated from the start of the recovery period (ZT 6), SD significantly reduced the latency to REM sleep in NAB mice compared to what was observed during baseline conditions from the start of sleep in the beginning of the light period. The latencies for both NREM and REM sleep were extremely reduced in LAB mice after SD when compared to baseline recordings; however, sleep latencies were not significantly altered by SD in HAB mice.

## Discussion

The present study examined the impact of trait anxiety on sleep-wake regulation. Specifically, sleep was differentially structured in mice with high (HAB) or low (LAB) levels of inborn anxiety-related behavior. HAB mice spent a greater part of their active (dark) phase asleep and had difficulty in maintaining long wake episodes. Strikingly, increased sleep in the dark period was accompanied by more frequent episodes of REM sleep. Moreover, HAB mice exhibited remarkably unstable ultradian cycles of sleep and an overall increase in delta activity during NREM sleep. In contrast, LAB mice showed longer sleep latencies, a significant increase in beta activity during NREM sleep and extensive episodes of wakefulness resulting in a reduction of the total amount of sleep.

The resting period of HAB mice was highly fragmented. Sleep fragmentation is a notable correlate of many affective disorders, including depression [34]–[36] and post-traumatic stress disorder [37], [38]. The prevalence of insomnia ranges from 70% to 90% in patients with anxiety disorders [39]. Patients with generalized anxiety disorder (GAD) frequently report difficulties in staying asleep, restlessness, and non-refreshing sleep [40], [41]. Many studies have also reported sleep disruption, associated with increased arousal at nighttime, as a feature of anxiety [42]–[44]. However, it was shown that, although GAD is characterized by physiological states of hyperarousal, objective findings on the polysomnogram often cannot be distinguished from those of healthy volunteers without sleep problems [45]–[48]. We expected to find numerous short arousals in HAB mice that could explain impaired sleep continuity during the light period but, surprisingly, the number of awakenings was not significantly increased. Rather, we found extremely short and frequent episodes of NREM and REM sleep to be responsible for the less stable pattern of sleep observed in HAB mice. Due to rapid shifts from NREM sleep to REM sleep and *vice versa,* HAB mice displayed a disturbed distribution of sleep stages and therefore an altered continuity of sleep. In general, it is difficult to ascertain cause-effect relationships between anxiety disorders and sleep complaints in patients. The present study demonstrates, for the first time, that the diminished capacity to sustain ultradian cycles of NREM and REM sleep, combined with an unconsolidated distribution of vigilance states, are sleep features that accompany elevated levels of inborn anxiety.

In contrast to hypersomniac patterns with wake-drive deficits in HAB mice, LAB mice showed longer sleep latencies and extensive wake episodes that resemble human insomnia. At the beginning of the resting period, LAB mice seemed incapable of down-regulating their arousal level. Moreover, they displayed marked increases in cortical beta activity during NREM sleep. Such malfunction of wake regulation resembles patients with primary insomnia accompanied by hyperarousal, circadian dysrhythmia and increased EEG activity across high frequency bands [49]. It remains to be shown to which extent LAB mice might represent a model for primary insomnia.

During the dark period, HAB mice spent remarkably more time in NREM and REM sleep. As a result, they showed decreased wakefulness at a time when mice, as nocturnal animals, would normally be spending more time awake. Hypersomnia (increased daytime sleepiness) has been described in subjects with increased anxiety [50]–[55]. According to the International Classification of Sleep Disorders, idiopathic (primary) hypersomnia is associated with excessive sleepiness, consisting of prolonged sleep episodes of NREM sleep [56]. Interestingly, the length of NREM and REM bouts in HAB mice was longer during the dark period than during the light period. In addition to this very unique phenomenon, a large proportion of sleep episodes seemed to pass prematurely from NREM to REM sleep. Furthermore, changes from wake to sleep states and *vice versa* were also frequently observed in HAB mice. Although anxiety disorders are reportedly linked to excessive daytime sleepiness [57], [58], it is difficult to evaluate whether this applies to HAB mice or whether their capacity of maintaining wakefulness is impaired.

We speculate that the inability of HAB mice to sustain persistent wakefulness and also to disinhibit frequent REM sleep intrusions may be a consequence of altered control of arousal-related brain regions. It is well documented that the dorsomedial-perifornical hypothalamus, which is enriched in neurons expressing hypocretin (also known as orexin), plays a crucial role in arousal and REM sleep inhibition [59]–[62]. Given the fact that the hypocretin system is also implicated in the development of anxiety disorders and depression [63], [64], examining hypocretinergic transmission in HAB mice would be an interesting point for future studies.

Another characteristic of the altered sleep parameters in HAB mice was a dramatic increase in EEG delta power during the entire light-dark cycle. Similar to HAB mice, increased EEG delta power during NREM sleep is found in patients with sleep-maintenance insomnia [65], manifested as sleep fragmentation and non-restorative sleep [66]. Delta power during NREM sleep reflects a need or a pressure for NREM sleep [67]–[69], suggesting that the enhanced sleep requirement in HAB mice may be the result of altered sleep regulation caused by high anxiety. Changes in EEG delta power during NREM sleep reflect sleep homeostasis and restorative functions of sleep [70]–[72]. In both humans and rodents, long period of wakefulness is followed by higher-than-normal EEG delta power at the beginning of NREM sleep, and thereafter the power gradually declines over the course of sleep [67], [73]–[76]. Given the disturbed sleep and waking patterns in HAB mice, we expected that the amplitude of delta oscillations during NREM sleep would be affected. Sleep continuity is important to restore the benefit of sleep [77]–[79]. Therefore, HAB mice were never able to fully restore their need to sleep. Consequently, the increased demand for sleep in HAB mice must have resulted from their inability to maintain the full (comprehensive) length of NREM sleep, thereby preventing a decline in sleep pressure.

In contrast to HAB mice, homeostatic responses, e.g., after SD, were rarely displayed by LAB mice. SD causes a short-lasting intensification of sleep, as indicated by the enhanced EEG delta power during NREM sleep. However, it is well documented that the duration of the prior wake period directly influences succeeding sleep bouts, especially with the sleep rebound [67], [73], [75], [80]. We speculate that a prolonged waking episode in the beginning of the resting period in LAB mice did not efficiently allow sleep pressure to accumulate and thereby these phenomena interfered with a process of homeostatic sleep control.

HAB mice showed diminished nocturnal activity, i.e., a flattened 24 h pattern of sleep-wake cycles; on the other hand, LAB mice tended to show a delay in the timing of sleep. This indicates that the amount, timing, and robustness of sleep-wake cycles are dysregulated in mice with different genetic predispositions to anxiety-related behavior. The majority of the diurnal aspects of behavior and physiology is coordinated by clock genes in the suprachiasmatic nucleus (SCN), which harbors the master circadian clock [81], [82]. Depending on the circadian phase, the circadian clock may actively promote sleep or wakefulness [83], [84]. A large body of literature indicates a significant link between the functioning of clock genes and behavioral changes that are directly related to affective disorders [85]–[92]. Recently, a human association study revealed that genes contributing to circadian rhythms might also play a role in the genetic predisposition to anxiety disorders [93]. Further, we recently found that the SCN protein levels of PER2, a reliable tissue clock marker [94], are lower in HAB than LAB mice (unpublished data), indicating that inborn differences in anxiety-related behavior may affect the intradiem oscillations of clock genes, leading to disrupted rhythmic day-night variations in the sleep-wake cycle.

There have been reports on altered sleep-wake modulation in other animal models of depression that were generated similarly by selective breeding within the CD1 mouse strain in order to investigate different aspects of psychopathology. For example, mice selected from long duration immobility in the tail suspension test (TST), that indicates high spontaneous helplessness (HL), are characterized by lighter slow wave sleep, increased REM sleep, and sleep fragmentation [14], [15]. Another example, the highly stress reactive (HR) mice based upon hypothalamic-pituitary-adrenal axis hyperreactivity in response to restrain stress, displays reduced sleep efficacy and increased REM sleep [8], [13]. However, while sharing common features in their sleep phenotype, these models showed some discrepancies in their behavioral traits. Compared to increased anxiety-related behavior of the HAB line, no differences in trait anxiety were revealed in HR mice [95]. While HL was reported to exhibit enhanced depression-like behavior in response to the TST and forced swimming [14], neither HAB nor HR animals clearly showed this passive coping style when compared with their corresponding controls [18], [95]. The behavioral differences across CD1 models thus indicate that selection for a particular trait resulted in changes in several sleep readouts, likely *via* different mechanisms underlying anxiety- and/or depression-related indices, with HAB mice being predominantly driven by trait anxiety.

Based on evidence of a strong genetic influence on sleep architecture [96] and well-described sleep disruptions associated with mood disorders [97]–[99], it was previously suggested that both awake and sleep EEG can serve as biomarkers of these psychiatric diseases [100]–[103]. The aim of this study was to define specific sleep characteristics that might help to distinguish a subpopulation of anxiety patients and thus allow for more specific determination of a psychopathological entity. Although primarily bred for extremes in trait anxiety, HAB and LAB mice also differ in passive vs. active stress coping, the former being indicative of depression-like behavior [18]. This animal model thus simulates the complex human phenotype of hyper-anxiety and comorbid depression. Increased EEG delta activity, unstable NREM sleep, and enhanced affinity to REM sleep are the most reliable EEG measures specific to HAB mice, which represent a mixture derived from both elements of anxiety and depression. Similarly, the increased drive towards REM sleep, i.e., REM sleep disinhibition, is a frequently observed sleep characteristic in depression [104]. Indeed, those increased REM sleep and fragmented sleep patterns were similarly characterized not only in the aforementioned mouse models but also in validated rat models of depression [9]–[12]. Most of these models, however, do not share altered EEG spectra. Further, it is important to note that sleep-wake abnormalities in HAB mice occurred throughout the day cycle including the active period. Increased delta activity throughout 24 h was also robust and unique, which has never been reported in any other rodent model. Thus, sleep phenotyping has the potential to crucially complement the behavioral repertoire to better distinguish between anxiety- and depression-related components, and supports valid models for translational studies.

In summary, we demonstrate that the divergence of trait anxiety in HAB vs. LAB mice severely modifies the structure, dynamics, and homeostasis of sleep-wake behavior. LAB mice exhibited delayed sleeping periods and altered control of sleep-loss related homeostasis. Sleep abnormalities found in HAB mice included persistently high sleep pressure and sleep fragmentation due to the rapid progression from NREM to REM sleep states. Moreover, an increased prevalence of REM sleep and significant reductions in the length of wakefulness were characteristics of HAB mice during the active period. In view of the profound sleep phenotypes of mice with selective alterations in inborn anxiety, these animal models may resemble trait-related sleep impairments of patients with anxiety and comorbid depression, and may also be used to discover both the mechanisms and molecular targets contributing to highly comorbid sleep abnormalities as well as to screen novel psychotropic drugs with selective or dual action on both psychopathologies.

## References

[1] Wong ML, Licinio J (2004). From monoamines to genomic targets: a paradigm shift for drug discovery in depression.. Nat Rev Drug Discov.

[2] Benca RM, Okawa M, Uchiyama M, Ozaki S, Nakajima T (1997). Sleep and mood disorders.. Sleep Med Rev.

[3] Steiger A, Kimura M (2009). Wake and sleep EEG provide biomarkers in depression.. J Psychiatr Res.

[4] Tsuno N, Besset A, Ritchie K (2005). Sleep and depression.. J Clin Psychiatry.

[5] Baldwin DS, Papakostas GI (2006). Symptoms of fatigue and sleepiness in major depressive disorder.. J Clin Psychiatry.

[6] Brown TA, Barlow DH (1995). Long-term outcome in cognitive-behavioral treatment of panic disorder: clinical predictors and alternative strategies for assessment.. J Consult Clin Psychol.

[7] Brown CS (2001). Depression and anxiety disorders.. Obstet Gynecol Clin North Am.

[8] Fenzl T, Touma C, Romanowski CP, Ruschel J, Holsboer F (2011). Sleep disturbances in highly stress reactive mice: modeling endophenotypes of major depression.. BMC Neurosci.

[9] Adrien J, Dugovic C, Martin P (1991). Sleep-wakefulness patterns in the helpless rat.. Physiol Behav.

[10] Dugovic C, Solberg LC, Redei E, Van Reeth O, Turek FW (2000). Sleep in the Wistar-Kyoto rat, a putative genetic animal model for depression.. Neuroreport.

[11] Shiromani PJ, Overstreet D, Levy D, Goodrich CA, Campbell SS (1988). Increased REM sleep in rats selectively bred for cholinergic hyperactivity.. Neuropsychopharmacology.

[12] Vogel G, Neill D, Kors D, Hagler M (1990). REM sleep abnormalities in a new animal model of endogenous depression.. Neurosci Biobehav Rev.

[13] Touma C, Fenzl T, Ruschel J, Palme R, Holsboer F (2009). Rhythmicity in mice selected for extremes in stress reactivity: behavioural, endocrine and sleep changes resembling endophenotypes of major depression.. PLoS One.

[14] El Yacoubi M, Bouali S, Popa D, Naudon L, Leroux-Nicollet I (2003). Behavioral, neurochemical, and electrophysiological characterization of a genetic mouse model of depression.. Proc Natl Acad Sci U S A.

[15] Popa D, El Yacoubi M, Vaugeois JM, Hamon M, Adrien J (2006). Homeostatic regulation of sleep in a genetic model of depression in the mouse: effects of muscarinic and 5-HT1A receptor activation.. Neuropsychopharmacology.

[16] Shekhar A, McCann UD, Meaney MJ, Blanchard DC, Davis M (2001). Summary of a National Institute of Mental Health workshop: developing animal models of anxiety disorders.. Psychopharmacology (Berl).

[17] Uys JD, Stein DJ, Daniels WM, Harvey BH (2003). Animal models of anxiety disorders.. Curr Psychiatry Rep.

[18] Krömer SA, Kessler MS, Milfay D, Birg IN, Bunck M (2005). Identification of glyoxalase-I as a protein marker in a mouse model of extremes in trait anxiety.. J Neurosci.

[19] Landgraf R, Kessler MS, Bunck M, Murgatroyd C, Spengler D (2007). Candidate genes of anxiety-related behavior in HAB/LAB rats and mice: focus on vasopressin and glyoxalase-I.. Neurosci Biobehav Rev.

[20] Sartori SB, Hauschild M, Bunck M, Gaburro S, Landgraf R (2011). Enhanced fear expression in a psychopathological mouse model of trait anxiety: pharmacological interventions.. PLoS One.

[21] Neumann ID, Wegener G, Homberg JR, Cohen H, Slattery DA (2011). Animal models of depression and anxiety: What do they tell us about human condition?. Prog Neuropsychopharmacol Biol Psychiatry.

[22] Murgatroyd C, Wigger A, Frank E, Singewald N, Bunck M (2004). Impaired repression at a vasopressin promoter polymorphism underlies overexpression of vasopressin in a rat model of trait anxiety.. J Neurosci.

[23] Ditzen C, Jastorff AM, Kessler MS, Bunck M, Teplytska L (2006). Protein biomarkers in a mouse model of extremes in trait anxiety.. Mol Cell Proteomics.

[24] Czibere L, Baur LA, Wittmann A, Gemmeke K, Steiner A (2011). Profiling trait anxiety: transcriptome analysis reveals cathepsin B (Ctsb) as a novel candidate gene for emotionality in mice.. PLoS One.

[25] Filiou MD, Zhang Y, Teplytska L, Reckow S, Gormanns P (2011). Proteomics and metabolomics analysis of a trait anxiety mouse model reveals divergent mitochondrial pathways.. Biol Psychiatry.

[26] Zhang Y, Filiou MD, Reckow S, Gormanns P, Maccarrone G (2011). Proteomic and metabolomic profiling of a trait anxiety mouse model implicate affected pathways.. Mol Cell Proteomics 10: M111 008110.

[27] Ohl F, Toschi N, Wigger A, Henniger MS, Landgraf R (2001). Dimensions of emotionality in a rat model of innate anxiety.. Behav Neurosci.

[28] Franken P, Dijk DJ, Tobler I, Borbely AA (1991). Sleep deprivation in rats: effects on EEG power spectra, vigilance states, and cortical temperature.. Am J Physiol.

[29] Kimura M, Müller-Preuss P, Lu A, Wiesner E, Flachskamm C (2010). Conditional corticotropin-releasing hormone overexpression in the mouse forebrain enhances rapid eye movement sleep.. Mol Psychiatry.

[30] Louis RP, Lee J, Stephenson R (2004). Design and validation of a computer-based sleep-scoring algorithm.. J Neurosci Methods.

[31] Franken P, Malafosse A, Tafti M (1998). Genetic variation in EEG activity during sleep in inbred mice.. Am J Physiol.

[32] Baracchi F, Opp MR (2008). Sleep-wake behavior and responses to sleep deprivation of mice lacking both interleukin-1 beta receptor 1 and tumor necrosis factor-alpha receptor 1.. Brain Behav Immun.

[33] Jakubcakova V, Flachskamm C, Deussing JM, Kimura M (2011). Deficiency of corticotropin-releasing hormone type-2 receptor alters sleep responses to bacterial lipopolysaccharide in mice.. Brain Behav Immun.

[34] Jones D, Gershon S, Sitaram N, Keshavan M (1987). Sleep and depression.. Psychopathology.

[35] Perlis ML, Giles DE, Buysse DJ, Thase ME, Tu X (1997). Which depressive symptoms are related to which sleep electroencephalographic variables?. Biol Psychiatry.

[36] Perlis ML, Giles DE, Buysse DJ, Tu X, Kupfer DJ (1997). Self-reported sleep disturbance as a prodromal symptom in recurrent depression.. J Affect Disord.

[37] Mellman TA, Kulick-Bell R, Ashlock LE, Nolan B (1995). Sleep events among veterans with combat-related posttraumatic stress disorder.. Am J Psychiatry.

[38] Ohayon MM, Shapiro CM (2000). Sleep disturbances and psychiatric disorders associated with posttraumatic stress disorder in the general population.. Compr Psychiatry.

[39] Maher MJ, Rego SA, Asnis GM (2006). Sleep disturbances in patients with post-traumatic stress disorder: epidemiology, impact and approaches to management.. CNS Drugs.

[40] Anderson DJ, Noyes R, Crowe RR (1984). A comparison of panic disorder and generalized anxiety disorder.. Am J Psychiatry.

[41] American Psychiatric Association (1994). Diagnostic and Statistical Manual of Mental Disorders, Forth Edition. Washington, DC: American Psychiatric Association.. 886 p.

[42] Fuller KH, Waters WF, Binks PG, Anderson T (1997). Generalized anxiety and sleep architecture: a polysomnographic investigation.. Sleep.

[43] Staner L (2003). Sleep and anxiety disorders.. Dialogues Clin Neurosci.

[44] Ohayon MM, Roth T (2003). Place of chronic insomnia in the course of depressive and anxiety disorders.. J Psychiatr Res.

[45] Papadimitriou GN, Linkowski P (2005). Sleep disturbance in anxiety disorders.. Int Rev Psychiatry.

[46] Vgontzas AN, Chrousos GP (2002). Sleep, the hypothalamic-pituitary-adrenal axis, and cytokines: multiple interactions and disturbances in sleep disorders.. Endocrinol Metab Clin North Am.

[47] Krystal AD, Edinger JD, Wohlgemuth WK, Marsh GR (2002). NREM sleep EEG frequency spectral correlates of sleep complaints in primary insomnia subtypes.. Sleep.

[48] Uhde TW, Cortese BM, Vedeniapin A (2009). Anxiety and sleep problems: emerging concepts and theoretical treatment implications.. Curr Psychiatry Rep.

[49] Pigeon WR, Perlis ML (2006). Sleep homeostasis in primary insomnia.. Sleep Med Rev.

[50] Monti JM, Monti D (2000). Sleep disturbance in generalized anxiety disorder and its treatment.. Sleep Med Rev.

[51] Ohayon MM (2008). From wakefulness to excessive sleepiness: what we know and still need to know.. Sleep Med Rev.

[52] van Mill JG, Hoogendijk WJ, Vogelzangs N, van Dyck R, Penninx BW (2010). Insomnia and sleep duration in a large cohort of patients with major depressive disorder and anxiety disorders.. J Clin Psychiatry.

[53] Tan TL, Kales JD, Kales A, Soldatos CR, Bixler EO (1984). Biopsychobehavioral correlates of insomnia. IV: Diagnosis based on DSM-III.. Am J Psychiatry.

[54] Szelenberger W, Soldatos C (2005). Sleep disorders in psychiatric practice.. World Psychiatry.

[55] Ford DE, Kamerow DB (1989). Epidemiologic study of sleep disturbances and psychiatric disorders. An opportunity for prevention?. Jama.

[56] American Psychiatric Association Sleep disorders (2000). Diagnostic and Statistical Manual of Mental Disorders, Forth Edition, text revision. Washington, DC: American Psychiatric Press.. 551–607 p.

[57] Theorell-Haglow J, Lindberg E, Janson C (2006). What are the important risk factors for daytime sleepiness and fatigue in women?. Sleep.

[58] Hasler G, Buysse DJ, Gamma A, Ajdacic V, Eich D (2005). Excessive daytime sleepiness in young adults: a 20-year prospective community study.. J Clin Psychiatry.

[59] Mochizuki T, Crocker A, McCormack S, Yanagisawa M, Sakurai T (2004). Behavioral state instability in orexin knock-out mice.. J Neurosci.

[60] Carter ME, Borg JS, de Lecea L (2009). The brain hypocretins and their receptors: mediators of allostatic arousal.. Curr Opin Pharmacol.

[61] Sakurai T (2007). The neural circuit of orexin (hypocretin): maintaining sleep and wakefulness.. Nat Rev Neurosci.

[62] Anaclet C, Parmentier R, Ouk K, Guidon G, Buda C (2009). Orexin/hypocretin and histamine: distinct roles in the control of wakefulness demonstrated using knock-out mouse models.. J Neurosci.

[63] Johnson PL, Truitt W, Fitz SD, Minick PE, Dietrich A (2010). A key role for orexin in panic anxiety.. Nat Med.

[64] Scott MM, Marcus JN, Pettersen A, Birnbaum SG, Mochizuki T (2011). Hcrtr1 and 2 signaling differentially regulates depression-like behaviors.. Behav Brain Res.

[65] Besset A, Villemin E, Tafti M, Billiard M (1998). Homeostatic process and sleep spindles in patients with sleep-maintenance insomnia: effect of partial (21 h) sleep deprivation.. Electroencephalogr Clin Neurophysiol.

[66] Rosenberg RP (2006). Sleep maintenance insomnia: strengths and weaknesses of current pharmacologic therapies.. Ann Clin Psychiatry.

[67] Franken P, Chollet D, Tafti M (2001). The homeostatic regulation of sleep need is under genetic control.. J Neurosci.

[68] Dijk DJ, Beersma DG (1989). Effects of SWS deprivation on subsequent EEG power density and spontaneous sleep duration.. Electroencephalogr Clin Neurophysiol.

[69] Borbely AA, Achermann P (1999). Sleep homeostasis and models of sleep regulation.. J Biol Rhythms.

[70] Knoblauch V, Krauchi K, Renz C, Wirz-Justice A, Cajochen C (2002). Homeostatic control of slow-wave and spindle frequency activity during human sleep: effect of differential sleep pressure and brain topography.. Cereb Cortex.

[71] Borbely AA, Baumann F, Brandeis D, Strauch I, Lehmann D (1981). Sleep deprivation: effect on sleep stages and EEG power density in man.. Electroencephalogr Clin Neurophysiol.

[72] Franken P, Tobler I, Borbely AA (1991). Sleep homeostasis in the rat: simulation of the time course of EEG slow-wave activity.. Neurosci Lett.

[73] Tobler I, Borbely AA (1986). Sleep EEG in the rat as a function of prior waking.. Electroencephalogr Clin Neurophysiol.

[74] Tobler I, Borbely AA (1990). The effect of 3-h and 6-h sleep deprivation on sleep and EEG spectra of the rat.. Behav Brain Res.

[75] Tobler I, Jaggi K (1987). Sleep and EEG spectra in the Syrian hamster (Mesocricetus auratus) under baseline conditions and following sleep deprivation.. J Comp Physiol A.

[76] Franken P, Malafosse A, Tafti M (1999). Genetic determinants of sleep regulation in inbred mice.. Sleep.

[77] Bonnet MH (1985). Recovery of performance during sleep following sleep deprivation in older normal and insomniac adult males.. Percept Mot Skills.

[78] Stepanski E, Lamphere J, Badia P, Zorick F, Roth T (1984). Sleep fragmentation and daytime sleepiness.. Sleep.

[79] Stepanski EJ (2002). The effect of sleep fragmentation on daytime function.. Sleep.

[80] Achermann P, Dijk DJ, Brunner DP, Borbely AA (1993). A model of human sleep homeostasis based on EEG slow-wave activity: quantitative comparison of data and simulations.. Brain Res Bull.

[81] Belle MD, Diekman CO, Forger DB, Piggins HD (2009). Daily electrical silencing in the mammalian circadian clock.. Science.

[82] Challet E (2007). Minireview: Entrainment of the suprachiasmatic clockwork in diurnal and nocturnal mammals.. Endocrinology.

[83] Franken P, Dijk DJ (2009). Circadian clock genes and sleep homeostasis.. Eur J Neurosci.

[84] Mistlberger RE (2005). Circadian regulation of sleep in mammals: role of the suprachiasmatic nucleus.. Brain Res Rev.

[85] Wirz-Justice A (2006). Biological rhythm disturbances in mood disorders.. Int Clin Psychopharmacol.

[86] Bunney JN, Potkin SG (2008). Circadian abnormalities, molecular clock genes and chronobiological treatments in depression.. Br Med Bull.

[87] Mendlewicz J (2009). Disruption of the circadian timing systems: molecular mechanisms in mood disorders.. CNS Drugs.

[88] Yang S, Van Dongen HP, Wang K, Berrettini W, Bucan M (2009). Assessment of circadian function in fibroblasts of patients with bipolar disorder.. Mol Psychiatry.

[89] Barnard AR, Nolan PM (2008). When clocks go bad: neurobehavioural consequences of disrupted circadian timing.. PLoS Genet.

[90] Lamont EW, Legault-Coutu D, Cermakian N, Boivin DB (2007). The role of circadian clock genes in mental disorders.. Dialogues Clin Neurosci.

[91] McClung CA (2007). Circadian genes, rhythms and the biology of mood disorders.. Pharmacol Ther.

[92] Turek FW (2007). From circadian rhythms to clock genes in depression.. Int Clin Psychopharmacol.

[93] Sipila T, Kananen L, Greco D, Donner J, Silander K (2011). An association analysis of circadian genes in anxiety disorders.. Biol Psychiatry.

[94] Shieh KR (2003). Distribution of the rhythm-related genes rPERIOD1, rPERIOD2, and rCLOCK, in the rat brain.. Neuroscience.

[95] Touma C, Bunck M, Glasl L, Nussbaumer M, Palme R (2008). Mice selected for high versus low stress reactivity: a new animal model for affective disorders.. Psychoneuroendocrinology.

[96] Kimura M, Winkelmann J (2007). Genetics of sleep and sleep disorders.. Cell Mol Life Sci.

[97] Benca RM, Obermeyer WH, Thisted RA, Gillin JC (1992). Sleep and psychiatric disorders. A meta-analysis.. Arch Gen Psychiatry.

[98] Breslau N, Roth T, Rosenthal L, Andreski P (1996). Sleep disturbance and psychiatric disorders: a longitudinal epidemiological study of young adults.. Biol Psychiatry.

[99] Buysse DJ, Hall M, Tu XM, Land S, Houck PR (1998). Latent structure of EEG sleep variables in depressed and control subjects: descriptions and clinical correlates.. Psychiatry Res.

[100] Friess E, Modell S, Brunner H, Tagaya H, Lauer CJ (2008). The Munich vulnerability study on affective disorders: microstructure of sleep in high-risk subjects.. Eur Arch Psychiatry Clin Neurosci.

[101] Rao U, Hammen CL, Poland RE (2009). Risk markers for depression in adolescents: sleep and HPA measures.. Neuropsychopharmacology.

[102] Murck H, Nickel T, Kunzel H, Antonijevic IA, Schill J (2003). State markers of depression in sleep EEG: dependency on drug and gender in patients treated with tianeptine or paroxetine.. Neuropsychopharmacology.

[103] Bruder GE, Sedoruk JP, Stewart JW, McGrath PJ, Quitkin FM (2008). Electroencephalographic alpha measures predict therapeutic response to a selective serotonin reuptake inhibitor antidepressant: pre- and post-treatment findings.. Biol Psychiatry.

[104] Lauer CJ, Riemann D, Wiegand M, Berger M (1991). From early to late adulthood. Changes in EEG sleep of depressed patients and healthy volunteers.. Biol Psychiatry.

